# Clinical trials in myasthenia gravis

**DOI:** 10.1007/s00415-023-11903-y

**Published:** 2023-07-31

**Authors:** A. Mahmood, J. Hawken, N. P. Robertson

**Affiliations:** 1 Department of Neurology, University Hospital of Wales, Heath Park, Cardiff, CF14 4XN UK; 2 Division of Psychological Medicine and Clinical Neuroscience, Department of Neurology, Cardiff University, University Hospital of Wales, Heath Park, Cardiff, CF14 4XN UK

Myasthenia Gravis is a chronic autoimmune disease associated with autoantibodies that are directed against post synaptic acetyl choline receptors in 80–88%. Although in many cases initial diagnosis and management is relatively straightforward, myasthenia remains a life-long condition without cure. Current treatment strategies are directed towards symptomatic relief with cholinesterase inhibitors and, in a proportion of patients, suppression of the immune response with the use of steroids and/or longer-term immunosuppressant strategies including oral immunosuppressants, plasma exchange, intravenous immunoglobulin, thymectomy, etc., many of which have significant side effects. Around 50% of patients will not respond to conventional treatment and disease course is often unpredictable with life-threatening myasthenic crises affecting 15–20% of patients at least once during their lives. In addition to the burden of disease on the individual, it has also been estimated that the financial cost from hospital admissions alone as a result of myasthenia was more than €450 million per year in Europe in 2010, and over $500 million in the USA in 2013.

As a result, more effective treatment options that might work alone or in combination with existing therapies and are better tolerated and less disruptive for patients would be welcomed. This month’s journal club explores three studies, using different therapeutic targets trying to achieve these aims, all using largely similar methodology and endpoints. Unusually for our journal club the studies reported here have all been published in a single issue of Lancet Neurology, which now seems to be establishing itself as a key journal for high profile randomised clinical trials in neurological disease.

## Safety and efficacy of rozanolixizumab in patients with generalised myasthenia gravis (MycarinG): a randomised, double-blind, placebo-controlled, adaptive phase 3 study

The neonatal Fc receptor prevents the lysosomal degradation of IgG, which in turn increases the half-life of circulating pathogenic IgG autoantibodies in myasthenia gravis. The use of rozanolixizumab (MycarinG), a neonatal Fc receptor inhibitor was assessed in this randomised double-blind, placebo-controlled, adaptive phase 3 study. Rozanolixizumab is a humanised IgG4 monoclonal antibody which acts on the IgG binding region of FcRn and interferes with IgG salvage and recycling. The study enrolled 300 patients with generalised myasthenia gravis (Myasthenia Gravis Foundation of America (MGFA) class II–IVa) who were ≥ 18 years old. Additional inclusion criteria were acetylcholine receptor (AChR) or muscle-specific kinase (MuSK) autoantibody positivity, and a Myasthenia Gravis Activities of Daily Living (MG-ADL) score of ≥ 3.

Over a course of 6 weeks, 200 patients randomly received (1:1:1) a once per week subcutaneous infusion of 7 mg/kg [66 (33%)], or 10 mg/kg [67 (34%)] of rozanolixizumab or placebo [67 (34%)]. Primary efficacy endpoint was change from baseline to day 43 in MG-ADL score, assessed in the intention-to-treat population.

Reductions in MG-ADL score from baseline to day 43 were greater in the rozanolixizumab 7 mg/kg group (least-squares mean change – 3.37 [SE 0.49]) and in the rozanolixizumab 10 mg/kg group (− 3.40 [0.49]) than with placebo (− 0.78 [0.49]; for 7 mg/kg, least-squares mean difference – 2.59 [95% CI – 4.09 to – 1.25], p < 0.0001; for 10 mg/kg, − 2.62 [− 3.99 to – 1.16], p < 0.0001). Treatment emergent adverse events (TEAE) were experienced by 52 (81%) of 64 patients treated with rozanolixizumab 7 mg/kg, 57 (83%) of 69 treated with rozanolixizumab 10 mg/kg, and 45 (67%) of 67 treated with placebo. Most frequent TEAEs were headache, diarrhoea, and pyrexia. Five (8%) patients in the rozanolixizumab 7 mg/kg group, seven (10%) in the rozanolixizumab 10 mg/kg group, and six (9%) in the placebo group had a serious TEAEs. Clinical efficacy of rozanolixizumab appeared robust in both AChR and MuSK positive generalised myasthenia.

Comment: Rozanolixizumab potentially offers a new therapeutic strategy for poorly controlled generalised myasthenia, with meaningful reductions in disease specific disability scores. However, adverse event rates are high and the longer-term effects of selectively targeting the neonatal Fc receptor will need to be monitored over the longer term.

Bril V, et al. Lancet Neurol. 2023 May;22(5):383–394.

## Safety and efficacy of zilucoplan in patients with generalised myasthenia gravis (RAISE): a randomised, double-blind, placebo-controlled, phase 3 study

RAISE is a randomised, double-blind, placebo-controlled trial assessing safety and efficacy of zilucoplan in patients with (AChR) positivity. Zilucoplan is a 15-amino acid macrocyclic peptide complement C5 inhibitor which is a key component in the pathogenesis of AChR-positive myasthenia gravis. Cleavage of complement component C5 triggers binding to other complement components resulting in the formation of membrane attack complexes (terminal complement complex), damaging the structural integrity of the postsynaptic membrane of the neuromuscular junction, and impairing the neuromuscular signal transmission. Zilucoplan blocks C5 cleavage and prevents the formation of membrane attack complex.

In the RAISE trial, out of 239 screened patients, 174 (73%) were recruited aged between 18 and 74. Following randomisation 86 (49%) patients received 0.3 mg/kg daily subcutaneous self-injection, and a further 88 (51%) had placebo for a period of 12 weeks. A statistically significant reduction in MG-ADL scores from baseline to 12 weeks post zilucoplan injection in comparison with placebo was reported (least squares mean change – 4.39 [95% CI – 5.28 to – 3.50] vs – 2.30 [− 3.17 to – 1.43]; least squares mean difference – 2.09 [− 3.24 to – 0.95]; p = 0.0004). TEAEs occurred in 77% of the zilucoplan group and 70% of the placebo group. Injection-site bruising following zilucoplan occurred in 14 patients (16%), compared to 8 patients (9%) in the placebo group. No anaphylactic reactions were reported and no serious infections. However, one death occurred in each of the two treatment groups which were considered unrelated (COVID-19 comorbidities, cerebral haemorrhage).

Comment: This study presents some promising results for zilucoplan and offers possibilities as a rapid onset, self-injected treatment for generalised myasthenia gravis. In addition, it seems that it can also be used with concomitant intravenous immunoglobulin and plasma exchange when rescue therapy is needed, with no supplemental dosing required, in contrast to monoclonal antibody treatments. However, cost may be a major factor in the availability of this treatment.

Howard JF Jr, et al. Lancet Neurol. 2023 May;22(5):395–406.

## Safety and clinical activity of autologous RNA chimeric antigen receptor T-cell therapy in myasthenia gravis (MG-001): a prospective, multicentre, open-label, non-randomised phase 1b/2a study

The third clinical trial reported in this edition of Lancet Neurology is MG-001; a USA-based multicentre-open-label, non-randomised phase 1b/2b trial. This study focussed on the safety and efficacy of autologous RNA chimeric antigen receptor T-cell therapy (rCAR-T) in managing myasthenia gravis. CAR-T is most commonly used in the management of haematological malignancies but issues of toxicity and lymphodepletion limits its use in myasthenia gravis and other autoimmune diseases. To overcome this, an engineered RNA (rCAR-T), Descartes-08, an autologous anti-BCMA rCAR-T agent was used to reduce potential toxicity and forgo the need for lymphodepletion.

A total of 14 participants with generalised myasthenia aged ≥ 18 were enrolled (10 females and 4 males) from eight USA centres with MG-ADL scores of ≥ 6. In phase 1b, 3 patients with MGFA disease class III–IV had three incremental doses of Descartes-08 to establish a maximum tolerated dose. Subsequently, 11 patients with MGFA class II–IV had 6 doses for 6 weeks at the maximum tolerated dose in an outpatient setting. Median follow-up was 5 months (range 3–9 months). MG-ADL scores improved from baseline to week 12, mean − 6 (95% CI − 9 to − 3), Quantitative Myasthenia Gravis score − 7 (− 11 to − 3), Myasthenia Gravis composite score − 14 (− 19 to − 9), and Myasthenia Gravis Quality of life 15-revised score − 9 (− 15 to − 3). No dose-limiting toxicity, cytokine release syndrome or neurotoxicity adverse events were reported. Two patients stopped their requirement for intravenous immunoglobulin infusion following treatment and overall participants demonstrated sustained clinical improvement which was well maintained for a year.

Comment: Preliminary results are certainly encouraging, and a more rigorous assessment of Descartes-08 clinical efficacy is ongoing in a randomised, placebo-controlled trial (NCT04146051). This trial could also pave the way for the use of rCAR-T therapy in other autoimmune diseases.

Granit V, et al. Lancet Neurol. 2023 Jul;22(7):578–590.

Summary: These three trials provide optimism for a new generation of clinically effective, well-tolerated therapies that could be used in conjunction with current standard treatments or as an alternative, with considerable potential for a more patient-tailored therapeutic approach. A more complete understanding of longer-term safety profiles of these drugs in myasthenia is yet to emerge but might potentially offer a lower burden of monitoring and could be either self-administered or provided in the outpatient setting. When applied appropriately, they may have the potential to minimise the frequency of myasthenia gravis-related hospital admissions. Future trials with larger sample sizes and more diverse populations may help to refine indications for use.

